# Synthesis and Characterization of Novel Dimeric Ionic Liquids by Conventional Approaches

**DOI:** 10.3390/ijms9071207

**Published:** 2008-07-14

**Authors:** Kilivelu Ganesan, Yatimah Alias

**Affiliations:** Department of Chemistry, Faculty of Science, University of Malaya, Kuala Lumpur, 50603, Malaysia E-mail: yatimah70@um.edu.my

**Keywords:** Imidazolium salt, dimer, anion exchange, Soxhlet extraction, chemical shift

## Abstract

The ^1^H-NMR shifts of the imidazolium protons of some novel dimeric ionic liquids were examined in various deuterated solvents. Interactions between the solvent and the imidazolium salt of butyl substituted ionic liquids were observed to give higher chemical shifts than methyl substitution.

## 1. Introduction

Ionic liquids are liquid at or near the room temperature; they can also be considered organic salts. Researchers have recently found that such ionic liquids are very useful as solvents as well as catalysts for several organic and inorganic syntheses and in polymer chemistry [1–4]. Ionic liquids are good candidates for replacement of toxic and volatile organic compounds because of their lower vapour pressures and lack of flammability [5–7]. Most organic solvents are unsuitable for high temperature organic reactions, while dicationic ionic liquids are more suitable because of their higher thermal stability and recyclability [8]. The imidazolium and pyridinium ions have played several crucial roles in the development of ionic liquid chemistry. Using a Green Chemistry approach Handy *et al*. synthesized a room temperature ionic liquid that acts as a catalyst for Heck reactions from monosugars [9]. Ionic liquids gave excellent yields in the ambient temperature conversion of carbonyl compounds into the respective alcohols with Grignard reagents [10]. Fujuta and coworkers synthesized various cyclic urethanes from amino alcohols and carbon dioxide using an ionic liquid as a catalyst in the presence of K_2_CO_3_ as promoter [11]. Synthesis of imidazolium based crystalline dimers of ionic liquids with calamitic-calamitic, calamitic-discotic, discotic-discotic moieties using microwaves has been reported [12]. Changing the anionic counterparts among BF_4_, SbF_6_, OTf, PF_6_, and NTf_2_ for 1,3-disubstituted imidazolium based ionic liquids plays an important role in the area of physical properties such as solubility, melting point and polarity [13–18]. Suarez *et al*. reported the reaction between dialkyl substituted imidazolium chloride with niobium pentachloride to give ionic mixtures whose properties depend upon the niobium concentration [19]. Recently novel multiply charged biradicals were synthesized from methylene bridged multiply charged imidazolium cations. These cations could function as gaseous precursors of suitable probes of the intrinsic reactivity of multiply charged radicals [20]. To the best of our knowledge, the sensitivity of the interactions between dimeric imidazolium salts and various solvents has not been reported so far. Herein, we wish to report the synthesis and characterization of novel dimeric imidazolium salts and examine the sensitivity of their interactions with deuterated solvents.

### 2. Results and Discussion

The synthesis of ionic liquids with 1,4-bis methylenebenzene as a linker unit started from the reaction of commercially available α,α′-dibromo-*p*-xylene with 2.1 equivalents of 1-methyl/1-butyl imidazole in the presence of dry CH_3_CN at room temperature with stirring for 24 hours to afford a white precipitate. The solvent was evaporated under reduced pressure and then the residue was washed several times with acetone to remove unreacted starting materials and give the imidazolium salts **2a**/**2e** as white solids in excellent yield (92–95 %).

The ^1^H-NMR spectrum of compound **2a** showed the two sharp singlets at δ 3.86 and 5.49 for the methyl and methylene protons, respectively. The phenyl protons appeared as a singlet at δ 7.51. Compound **2a** has three different protons in the imidazole moiety, and showed one singlet and two doublets at δ 9.42, 7.85 and 7.71, respectively. The ^13^C-NMR spectrum of compound **2a** showed signals at δ 35.3 and 51.3 for methyl and methylene carbons. Aromatic and imidazole carbons appeared in their respective region. Satisfactory elemental analysis further confirmed our proposed product. Similarly, butyl dicationic ionic liquid **2e** was fully characterized by spectral and analytical data.

The ionic liquids **2a**/**2e** underwent a metathesis reaction with a slight stoichiometric excess of NaBF_4_/KPF_6_/LiCF_3_SO_3_ (MX) in ethanol/water (4:1) ratio at room temperature for 4 hours. After completion of the reaction, solvents were removed under reduced pressure. Attempted extraction with various solvents like CHCl_3_, CH_2_Cl_2_ and EtOAc did not extract any ionic liquid from the organic layer because of their poor solubility in the the abovementioned organic solvents. Hence, a Soxhlet extractor was used to remove the inorganic salt. Using Soxhlet extraction with ethanol afforded the ionic liquids **2b-d**,**f-h** in 82–90 % yields.

Chemicals shift for the protons of the eight synthesized dimeric imidazolium ionic salts containing methyl/butyl substitution with various anions were recorded in different deuterated solvents (Tables 1 and 2). The dimeric materials are insoluble in some of the deuterated solvents like acetone, acetonitrile, ethyl acetate and chlorinated solvents.

We recorded the NMR spectra in different deuterated solvents with the same concentrations of ionic liquids **2a-h**; interestingly, we found that the chemical shift values were remarkable changed from TFA to DMF. We observed that acidic proton of the imidazolium dication shifted towards downfield depending on the deuterated solvent used as well as the anions. We employed four different anions, namely Br^−^, BF_4_^−^, PF_6_^−^ and CF_3_SO_3_^−^; chemical shift values of acidic protons appeared to follow the order Br^−^<BF_4_^−^<PF_6_^−^<CF_3_SO_3_^−^. This might be due to electronegativity as well as the bulkiness of the anions. As a result, the CF_3_SO_3_^−^ and PF_6_^−^ ions are better hydrogen bond acceptors than the Br^−^ or BF_4_^−^ salts. The imidazolium ring contains three different protons, namely H_2_, H_4_ and H_5_. The H_2_ proton is more sensitive than the other two protons because it is surrounded by two electronegative atoms; when the alkyl side chain as well as anions was changed interesting trends in the chemical shifts were observed.

## 3. Conclusions

The chemical shifts for the imidazolium protons of novel ionic liquids in various deuterated solvents were examined. We observed that acidic protons of the imidazolium dications are shifted from upfield, depending on both the anions and deuterated solvents. Interactions between the solvent and imidazolium salt of butyl substituted ionic liquids are observed higher chemical shifts than in methyl substituted ones.

## 4. Experimental Section

### 4.1. General

α,α′-Dibromo-*p*-xylene, 1-methyl imidazole, 1-butyl imidazole and solvents were purchased from commercial sources and used without further purification. ^1^H- and ^13^C-NMR were recorded on a Jeol 400 MHz NMR spectrometer operating at 400 and 100 MHz, respectively. Chemical shifts are reported relative to TMS as reference for proton and carbon. Elemental analyses were carried out on a ThermoFinnigan Eager 300 elemental analyzer.

### 4.2. General Procedure for the Synthesis of Dicationic Ionic Liquids

Compounds **2a**/**2e** were prepared by stirring one equivalent of α,α′-dibromo-*p*-xylene and 2.1 equivalents of methyl/butyl imidazole in dry CH_3_CN for 24 hours at room temperature to give the respective quaternized salts. Solvent was removed under reduced pressure and the residue washed with acetone to remove the unreacted imidazole, followed by filtration to afford white precipitates of the dicationic imidazolium salts **2a**/**2e** in quantitative yield. Compounds **2a**/**2e** were dissolved with 4:1 (ethanol/water) along with a slight molar excess of NaBF_4_/KPF_6_/LiCF_3_SO_3_ and the mixture was stirred for 4 hours at room temperature to afford the respective anion exchanged products **2b-d**, **f-h.** After completion of the reaction period, the mixture was concentrated under reduced pressure as much as possible, followed by addition of anhydrous MgSO_4_. The solid material was extracted using Soxhlet extraction with ethanol for 3 hours to afford the ionic liquids **2b-df-h**, in 82–90 % yield.

*1, 1′-(1,4-Phenylenebismethylene)bis(3-methyl-1H-imidazolium-1-yl) di(bromide)* (**2a**): Colorless solid; mp: 180 – 182 °C; Yield: 95 %; ^1^H-NMR (DMSO-*d_6_*): δ_H_ 9.42 (s, 2H), 7.85 (d, 2H), 7.71 (d, 2H), 7.51 (s, 4H), 5.49 (s, 4H), 3.86 (s, 6H); ^13^C-NMR: δ_C_ 136.3, 135.5, 128.6, 122.5, 122.0, 51.3, 35.3; Anal. Cald. (%) C_16_H_20_Br_2_N_4_ (428): C, 44.85; H, 4.67; N, 13.09. Found: C, 44.81; H, 4.62; N, 13.03.

*1,1′-(1,4-Phenylenebismethylene)bis(3-methyl-1H-imidazolium-1-yl) di(tetrafluoroborate)* (**2b**): Colorless solid; mp: 68 – 71 °C; Yield: 90 %; ^1^H-NMR (DMSO-*d_6_*): δ_H_ 9.48 (s, 2H), 7.87 (d, 2H), 7.74 (d, 2H), 7.49 (s, 4H), 5.53 (s, 4H), 3.89 (s, 6H); ^13^C-NMR: δ_C_ 136.5, 135.5, 128.7, 122.3, 122.4, 51.6, 35.5; Anal. Cald. (%) C_16_H_20_B_2_F_8_N_4_ (441): C, 43.53; H, 4.53; N, 12.69. Found: C, 43.49; H, 4.48; N, 12.55.

*1,1′-(1,4-Phenylenebismethylene)bis(3-methyl-1H-imidazolium-1-yl) di(hexafluorophosphate)* (**2c**): Liquid; Yield: 83 %; ^1^H-NMR (DMSO-d_6_): δ_H_ 9.55 (s, 2H), 7.86 (d, 2H), 7.75 (d, 2H), 7.50 (s, 4H), 5.48 (s, 4H), 3.87 (s, 6H); ^13^C-NMR: δ _C_ 136.6, 135.7, 128.3, 122.7, 122.4, 51.7, 35.7; Anal. Cald. (%) C_16_H_20_P_2_F_12_N_4_ (557.9): C, 34.41; H, 3.58; N, 10.30. Found: C, 34.38; H, 3.52; N, 10.28.

*1,1′-(1,4-Phenylenebismethylene)bis(3-methyl-1H-imidazolium-1-yl) di(trifluromethanesulphonate)* (**2d**): Liquid; Yield: 86 %; ^1^H-NMR (DMSO-*d_6_*): δ_H_ 9.59 (s, 2H), 7.90 (d, 2H), 7.77 (d, 2H), 7.50 (s, 4H), 5.48 (s, 4H), 3.87 (s, 6H); ^13^C-NMR: δ_C_ 136.8, 135.7, 128.8, 122.7, 122.4, 51.6, 35.8; Anal. Cald. (%) C_18_H_20_O_6_F_6_N_4_S_2_ (565.99): C, 38.16; H, 3.53; N, 9.89. Found: C, 38.12; H, 3.50; N, 19.85.

*1,1′-(1,4-Phenylenebismethylene)bis(3-butyl-1H-imidazolium-1-yl) di(bromide)* (**2e**): Colorless solid; mp: 139 – 142 °C; Yield: 92 %; ^1^H-NMR (DMSO-*d_6_*): δ_H_ 9.35 (s, 2H), 7.79 (d, 2H), 7.53 (d, 2H), 7.49 (s, 4H), 5.47 (s, 4H), 4.19 (t, 4H), 1.78 (m, 4H), 1.18 (m, 4H), 0.94 (t, 6H); ^13^C-NMR: δ_C_ 136.1, 135.4, 128.9, 122.8, 122.4, 51.3, 48.6, 31.2, 18.7, 13.2; Anal. Cald. (%) C_22_H_32_Br_2_N_4_ (512): C, 51.56; H, 6.25; N, 10.93. Found: C, 51.51; H, 6.20; N, 10.89.

*1,1′-(1,4-Phenylenebismethylene)bis(3-butyl-1H-imidazolium-1-yl) di(tetrafluroborate)* (**2f**): Liquid; Yield: 86 %; ^1^H-NMR (DMSO-*d_6_*): δ_H_ 9.44 (s, 2H), 7.82 (d, 2H), 7.77 (d, 2H), 7.49 (s, 4H), 5.45 (s, 4H), 4.17 (t, 4H), 1.74 (m, 4H), 1.21 (m, 4H), 0.89 (t, 6H); ^13^C-NMR: δ_C_ 136.4, 135.7, 128.8, 122.7, 122.1, 51.1, 48.4, 31.1, 18.5, 13.1; Anal. Cald. (%) C_22_H_32_B_2_F_8_N_4_ (525.6): C, 50.22; H, 6.08; N, 10.65. Found: C, 50.20; H, 6.03; N, 10.61.

*1,1′-(1,4-Phenylenebismethylene)bis(3-butyl-1H-imidazolium-1-yl) di(hexaflurophosphate)* (**2g**): Liquid; Yield: 84 %; ^1^H-NMR (DMSO-*d_6_*): δ_H_ 9.49 (s, 2H), 7.81 (d, 2H), 7.78 (d, 2H), 7.44 (s, 4H), 5.45 (s, 4H), 4.17 (t, 4H), 1.75 (m, 4H), 1.22 (m, 4H), 0.88 (t, 6H); ^13^C-NMR: δ_C_ 136.3, 135.7, 128.8, 122.6, 122.5, 51.5, 48.7, 31.5, 18.9, 13.7; Anal. Cald. (%) C_22_H_32_P_2_F_12_N_4_ (641.92): C, 41.12; H, 4.98; N, 8.72. Found: C, 41.09; H, 4.94; N, 8.67.

*1,1′-(1,4-Phenylenebismethylene)bis(3-butyl-1H-imidazolium-1-yl) di(trifluromethanesulphonate)* (**2h**): Colorless solid; mp: 59 – 62 °C; Yield: 82 %; ^1^H-NMR (DMSO-*d_6_*): δ_H_ 9.54 (s, 2H), 7.85 (d, 2H), 7.79 (d, 2H), 7.49 (s, 4H), 5.45 (s, 4H), 4.17 (t, 4H), 1.75 (m, 4H), 1.18 (m, 4H), 0.91 (t, 6H); ^13^C-NMR: δ_C_ 136.2, 135.8, 128.7, 122.9, 122.6, 51.5, 48.9, 31.5, 18.4, 13.5; Anal. Cald. (%) C_22_H_32_O_6_F_6_N_4_S_2_ (625.99): C, 42.17; H, 5.11; N, 8.94. Found: C, 42.14; H, 5.07; N, 8.90.

## Figures and Tables

**Scheme 1.**
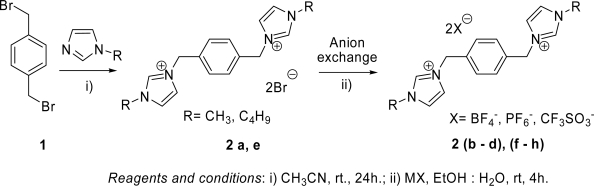
*Reagents and conditions*: i) CH_3_CN, rt., 24h.; ii) MX, EtOH: H_2_O, rt, 4h.

**Table 1.** Chemical shift values of methyl substituted dimeric salts in different deuterated solvents at 25°C.

**Figure:**
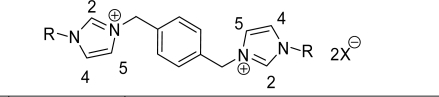

S. No	Solvent	Compound		δ H_2_	δ H_4_	δ H_5_
	1	TFA-d_1_	Br^−^	**2a**	8.58	7.74	7.28
			BF_4_^−^	**2b**	8.64	7.76	7.31
			PF_6_^−^	**2c**	8.74	7.78	7.29
			CF_3_SO_3_^−^	**2d**	8.78	7.75	7.32	
2	HOAc-d_4_	Br^−^	**2a**	8.68	7.76	7.30		
		BF_4_^−^	**2b**	8.76	7.77	7.35		
		PF_6_^−^	**2c**	8.84	7.81	7.32		
		CF_3_SO_3_^−^	**2d**	8.86	7.83	7.37	
3	MeOH-d_4_	Br^−^	**2a**	9.06	7.84	7.76	
		BF_4_^−^	**2b**	9.10	7.84	7.76	
		PF_6_^−^	**2c**	9.17	7.62	7.58	
		CF_3_SO_3_^−^	**2d**	9.23	7.84	7.76	
4	DMSO-d_6_	Br^−^	**2a**	9.42	7.85	7.71	
		BF_4_^−^	**2b**	9.48	7.87	7.74	
		PF_6_^−^	**2c**	9.55	7.86	7.75	
		CF_3_SO_3_^−^	**2d**	9.59	7.90	7.77	
5	DMF-d_7_	Br^−^	**2a**	9.64	8.00	7.87	
		BF_4_	**2b**	9.67	8.02	7.90	
		PF_6_^−^	**2c**	9.70	8.03	7.89	
		CF_3_SO_3_^−^	**2d**	9.76	8.03	7.90	
6	CD_3_CN	Compounds **2a**-**d** are insoluble					
7	Acetone-d_6_						
8	CDCl_3_							

**Table 2.** Chemical shift values of butyl substituted dimeric salt in various deuterated solvents at 25°C.

S. No	d-Solvent	Compound		δ H_2_	δ H_4_	δ H_5_
1	TFA-d_1_	Br^−^	**2e**	8.58	7.75	7.32
		BF_4_^−^	**2f**	8.63	7.73	7.30
		PF_6_^−^	**2g**	8.68	7.74	7.34
		CF_3_SO_3_^−^	**2h**	8.70	7.70	7.31
2	HOAc-d_4_	Br^−^	**2e**	8.74	7.74	7.29
		BF_4_^−^	**2f**	8.81	7.77	7.30
		PF_6_^−^	**2g**	8.79	7.80	7.33
		CF_3_SO_3_^−^	**2h**	8.84	7.82	7.39
3	MeOH-d_4_	Br^−^	**2e**	9.09	7.65	7.54
		BF_4_^−^	**2f**	9.11	7.66	7.62
		PF_6_^−^	**2g**	9.15	7.65	7.60
		CF_3_SO_3_^−^	**2h**	9.19	7.69	7.62
4	DMSO-d_6_	Br^−^	**2e**	9.35	7.79	7.53
		BF_4_^−^	**2f**	9.44	7.82	7.77
		PF_6_^−^	**2g**	9.49	7.81	7.78
		CF_3_SO_3_^−^	**2h**	9.54	7.85	7.79
5	DMF-d_7_	Br^−^	**2e**	9.62	8.01	7.95
		BF_4_^−^	**2f**	9.69	8.03	7.97
		PF_6_^−^	**2g**	9.77	8.06	7.98
		CF_3_SO_3_^−^	**2h**	9.98	8.09	8.02
6	CD_3_CN	Compound **2**(**e**-**h**) are insoluble				
7	Acetone-d_6_					
8	CDCl_3_					
